# Assessing the Role of Lizards as Potential Pollinators of an Insular Plant Community and Its Intraspecific Variation

**DOI:** 10.3390/ani13061122

**Published:** 2023-03-22

**Authors:** Víctor Romero-Egea, Cristina Robles, Anna Traveset, Laura Del Rio, Sandra Hervías-Parejo

**Affiliations:** 1 Department of Animal Health, Campus de Espinardo, University of Murcia, 30100 Murcia, Spainlaurario@um.es (L.D.R.); 2 Mediterranean Institute for Advanced Studies (IMEDEA, UIB-CSIC), Miquel Marquès 21, 07190 Esporles, Spain; 3 Centre for Functional Ecology (CFE-UC), Department of Life Sciences, University of Coimbra, Calçada Martim de Freitas, 3004-531 Coimbra, Portugal

**Keywords:** Balearic Islands, Cabrera archipelago, florivory, intra-specific resource use, plant–lizard interactions, *Podarcis lilfordi*, *Tarentola mauritanica*

## Abstract

**Simple Summary:**

The role of lizards as potential pollinators is increasingly recognized, especially on islands. However, there are very few studies at the community level that have also addressed intraspecific variations related to the consumption of floral resources. We pursued this objective on the island of Cabrera Gran (Balearic Islands) where there are the Balearic wall lizards (*Podarcis lilfordi*) and two geckos (*Tarentola mauritanica* and *Hemidactylus turcicus*). Balearic lizards have proven to be potential community-level pollinators by interacting with many different plant species to varying degrees. Although in some plant species lizards damaged reproductive structures by feeding directly on them, legitimate visits were significantly more frequent. Intraspecific differences were found in these wall lizards and even indications of gecko–flower interactions. These findings expand our knowledge not only on the magnitude of lizard–plant community interactions but also in their complexity.

**Abstract:**

The role of lizards as potential pollinators on islands has been documented for either one or a few plants in different parts of the world, but it has never been assessed for an entire plant community. Here, we quantified interaction rate by lizards and evaluated intraspecific differences in the use of flowers on Cabrera Gran (Cabrera archipelago, Balearic Islands) by means of visual observations, automated cameras and the analysis of pollen grain samples. Overall, we recorded interactions of the Balearic wall lizard (*Podarcis lilfordi*) with flowers of 44 plant species, 72.7% of which were unknown to date. Although florivory occurs in some of these species (35%), the majority of visits were legitimate (65%); in addition, we found intraspecific differences in the interactions related to the sex and age of lizards. Our findings support the role of Balearic wall lizards as potential pollinators across the entire plant community, and their contribution to particular plant species, for instance the endangered *Cistus heterophyllus carthaginensis*. This study also documents the first record of another sympatric lizard (*Tarentola mauritanica*) visiting flowers and contributes to the few existing records of flower interactions involving geckos in the Paleartic ecozone.

## 1. Introduction

Scaly reptiles (Squamata), named “lizards”, are typically characterized as predators that consume plant matter anecdotally [1,2,3], with the exception of some large species with adaptations to herbivorous life [4]. Therefore, it is not expected that these animals have relevant roles in plant reproduction through seed dispersal and pollination. This perception has changed greatly in recent decades, revealing more flexible trophic behaviors of lizards with important implications for the communities in which they are integrated [5,6,7,8,9,10].

The interactions of lizards with flowers have been documented in continental environments [3,11,12,13] but mostly on islands [1,5,9,14,15,16]. The low richness of species and, therefore, fewer interspecific competitors, parasites and predators, may explain the high densities reached by lizards on islands, due to the density compensation phenomenon [17]. These high densities of lizards, in areas where arthropods, which are their usual prey, are scarce, lead to a broadening of the trophic niche [18]. This is typical of islands, but also of isolated mainland ecosystems with similar environmental conditions to those on islands [19]. Specifically, the dietary changes that reptiles undergo, adapting the size of the resource to that of the individual, are relevant in these environments with high intraspecific competition [20,21,22]. In fact, different individuals of the same species may, depending on their specific needs, develop diverse trophic behaviors [23]. Moreover, sexual dimorphism has also been shown to affect ecomorphological diversity [24,25]. However, there are very few studies that have addressed intraspecific variations related to the consumption of floral resources [26,27,28,29,30].

Lizards that interact with flowers can damage the reproductive structures by feeding directly from them (i.e., florivory, 39.1% of all cases, as recently reviewed by Justicia Correcher et al. [19]. When visits are legitimate (i.e., come into contact with the reproductive organs of the flower without damaging them), lizards present low pollinating potential compared to other more mobile animals, such as birds, mammals and insects with better adhesion to pollen grains, and this can be a disadvantage. However, we now have evidence that lizards are not only legitimate but also effective visitors contributing to the pollination of plants on the islands [1,31,32,33,34]. The species of lizards and plants involved in these interactions, as well as the interactions themselves, are usually endemic to these unique sites. In any case, these studies generally focus only on particular species and/or interactions but not on the whole diversity of species of plants that are visited, or on the intraspecific differences between lizards of different ages and/or sex. It evidences the need to carry out studies at the community level on the lizard species that have already shown their potential as pollinators.

The Cabrera archipelago is, today, the largest territory of the Balearic lizard (*Podarcis lilfordi*), a lacertid that was originally largely distributed but is now restricted to the smaller islands. In areas where they have disappeared, the distribution of some lizard-depending plants for pollen and/or seed dispersal have undergone radical changes [35]. Although some lizard–flower interactions have been reported (see review in Justicia Correcher et al. [19]), the relationship of the Balearic lizard with most of the flora is still unknown. In this study, we used a novel approach to estimate the potential pollinator role of the Balearic lizard at the community level, including the floristic diversity of its trophic spectrum (possibly underestimated) and analyzing the relevance of legitimate visits versus those involving florivory. In addition, we studied the effect of age and sex on the interactions with flowers by this species. Thus, our objectives were (i) to identify and quantify plant–lizard interactions at the community level, and (ii) to assess intraspecific differences (i.e., between males, females and juveniles) in the type of interaction they establish with flowers. Furthermore, (iii) we explored the use of flowers as a trophic resource by other lizards present on the island.

## 2. Materials and Methods

### 2.1. Study Site and Species

Fieldwork was carried out on Cabrera Gran, the largest island of the archipelago (15.7 km^2^, 39°08′31″ N 2°56′45″ E), from 5 April to 6 May 2022, coinciding with the flowering of a good part of the vegetal community. The data were collected throughout the entire island based on flower availability.

Of the three lizards present in the Cabrera archipelago, the predominant species is the Balearic lizard, an endemic lacertid which spreads over all habitats, from extensive pine forests to minuscule islets without vegetation cover but seasonally occupied by colonies of seabirds [36]. The Balearic lizard consumes plant matter regularly [37,38], including both fruits [39,40,41,42,43,44] and floral resources [39,45,46,47]. Considering its entire current distribution (Cabrera archipelago, Dragonera and small islets of Mallorca and Menorca), it is known to interact with the flower of at least 59 plant species (51% legitimate visitation, 32% florivory), 21 (36%) of which have been reported from the Cabrera archipelago and 6 (10%; *Lavatera maritima*, *Globularia alypum*, *Ephedra fragilis*, *Euphorbia dendroides*, *Euphorbia characias* and *Fumana ericoides*) from the island of Cabrera Gran [19]. In addition to the Balearic lizard, two geckos with a wide distribution in the Mediterranean basin are found in Cabrera: the Moorish gecko, *Tarentola mauritanica*, and the Turkish gecko, *Hemidactylus turcicus*. Although there are no records of visiting flowers by these geckos (nor of any herbivorous behavior anywhere in the world), *Ficus carica* seeds have been found in stomach contents of Moorish geckos precisely in this island [48,49]. This species shares some habitats with Balearic lizards and even thermoregulate together (personal obs.).

### 2.2. Plant–Lizard Interactions

To document lizard–flower interactions, we used three sampling techniques: direct observation censuses, automatic cameras and pollen smears. The censuses consisted of 10 min observations, alternating different days, hours and areas, until completing 60 min per plant species, in daytime conditions of clear skies and little or no wind. In each census, we recorded the total number of flowers available, the number of flowers observed and the number of flowers visited, as well as the species, sex and age (i.e., adult or juvenile) of lizards and the type of the interaction (i.e., legitimate visit or florivory). Individual lizards were then grouped into one of three groups (males, females or juveniles) to account for intraspecific size differences that may have functional effects. Any flowering plant was censused (Supplementary File), although those with the greatest potential to be visited by vertebrates were prioritized, either due to their location (e.g., growing next to the walls, which are used by the lizards as shelter) or relative abundance. Endemicity or degree of threat was considered too. When 10 min observations could not be performed (e.g., environmental conditions were not optimal, floral species were short-lived, flowers or lizards were very scarce), non-systematic observations were carried out to determine at least the presence/absence of lizard–flower interactions. In addition, we used nine automatic cameras (8 MP V2 Module), controlled by a single-board computer (Raspberry Pi 4), programmed to make 5 min recordings (1080p/30 frames per second) with 3 min rest intervals. We placed the cameras in front of each flowering plant species and rotated them to different species and areas of the island to obtain the highest possible number of interactions per plant species. We processed the images manually (46 h of filming on 19 plant species) and recorded the same variables used in the censuses. Both direct observations and automated camera monitoring allow us to quantify the lizard–flower interactions. Thus, we obtained an interaction rate (IR) for each animal, estimated as the number of flowers contacted divided by the number of flowers observed and the observation time, and multiplied by the total number of flowers available. Moreover, in areas with high flower availability, an equal number of pollen samples were collected for each lizard group (i.e., males, females and juveniles) and geckos in order to detect interactions with flowering species that could not be recorded by either of the two previous techniques. Pollen samples provide valuable information on the importance of these animals as pollen dispersers. To do this, lizards were captured by manually grasping the animal by the neck, thus avoiding contact with the head (so as not to alter the original presence of pollen, if any) and the tail (so as not to trigger the caudal autotomy mechanism with the consequent energetic waste for the individual). For each animal captured, we recorded the sex and age and rubbed a small amount of glycerogelatine on the head (with special attention to areas prone to pollen contact, such as the corners of the mouth and muzzle) using tweezers. Before its release in the same place where it had been captured, the lizard was marked with a drop of white nail polish on its head to avoid recapture and, therefore, the collection of several samples from the same individual. The pollen sample was then fixed on a slide, melting the glycerogelatin with the flame of a lighter and sealing the coverslip with clear nail polish to avoid contamination of the sample. Finally, the pollen plates were examined under an optical microscope to identify the species, using the reference collection available at the IMEDEA. Only samples with more than six pollen grains of the same species or morphotype were considered, whereas less than six grains were considered possible contamination.

### 2.3. Representation of Species Interactions and Statistical Analyses

We constructed a binary quantitative ecological network, using data from surveys and automatic cameras, to illustrate the two types of interaction, using the IR values (i.e., the interaction weight) pooled into the three lizard groups (males, females and juveniles), with the ‘bipartite’ package in R [50].

We explored whether the frequency of legitimate visits versus florivory varies for each lizard group (males, females and juveniles), using a Pearson’s Chi-squared test with Yates’ continuity correction (package ‘CrossTable’ of the R ‘gmodels’ library [51]).

## 3. Results

**Figure 1:**
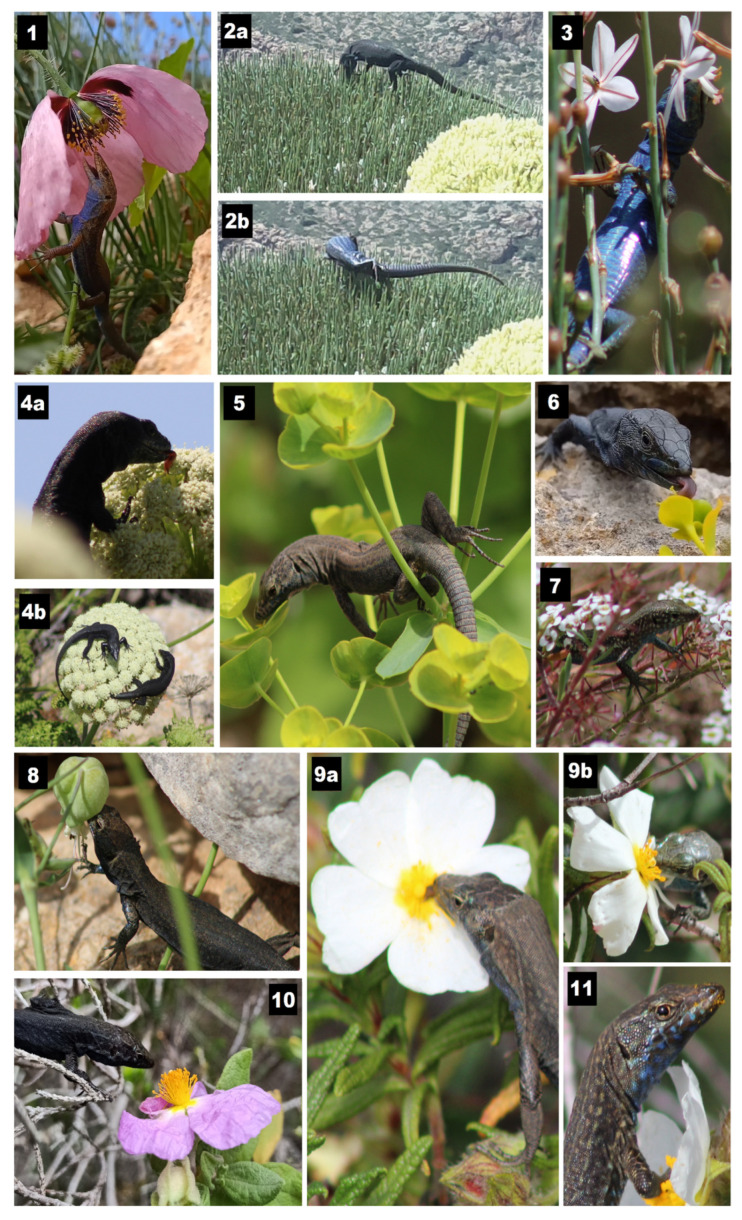
Lizard–flower interactions observed on Cabrera Gran: florivory in *Papaver somniferum* (**1**), *Astragalus balearicus* (**2**) and *Asphodelus fistulosus* (**3**); legitimate visits in *Daucus carota* (**4**), *Euphorbia segetalis* (**5**), *Euphorbia dendroides* (**6**), *Lobularia maritima* (**7**), Silene vulgaris (**8**), *Cistus monspeliensis* (**9**) and *Cistus heterophyllus* (**10**); and pollen grains visible over a lizard (**11**).

Through direct censuses, the analysis of recorded images, the identification of pollen samples and non-systematic observations we reported interactions between lizards and flowers of 44 plant species (n = 57), 41 of which are new to the island of Cabrera (Table 1; Figure 1). Overall, 72.7% of the species’ interactions with lizards had not been previously recorded. In total, 18% (n = 38) of lizard pollen samples contained pollen grains of *Plantago* sp., one Apiaceae species, and four morphospecies whose identification was not possible. The most frequently visited species (higher IR) were *Daucus carota*, *Euphorbia dendroides* and *Lobularia maritima*, followed by *Cistus monspeliensis*, *Lavatera arborea*, *Lomelosia cretica* and *Paronychia capitata*.

Lizards did not interact with all plant species in the same way; legitimate visits were more frequent (65% of the total number of species with observed interactions) than florivory (35%). Interactions of lizards with flowers were either casual (i.e., the lizard crawls over the exposed floral structures in its path), or intentional (i.e., the lizard searches for insects inside the flowers or feeds directly on them). In any case, we considered these interactions as legitimate visits as long as there was no damage to the reproductive organs of the flower. It could also be seen that some flowers were systematically bitten while in others the contact almost always occurred with the tongue. For example, while an infrequent encounter with the opium poppy (*Papaver somniferum*) produced a voracious attack, in other common species, such as euphorbias (*Euphorbia* spp.) or the jaguar (*Cistus monspeliensis*), a meticulous nectar-licking behavior was shown. The interaction with this last genus, not reported until now, was revealed as one of the most frequent in Cabrera. At noon, a multitude of lizards expose themselves to potential predators by climbing over the sage of this species to collect nectar and pollen with their tongues, moving from one flower to another.

**Figure 2:**
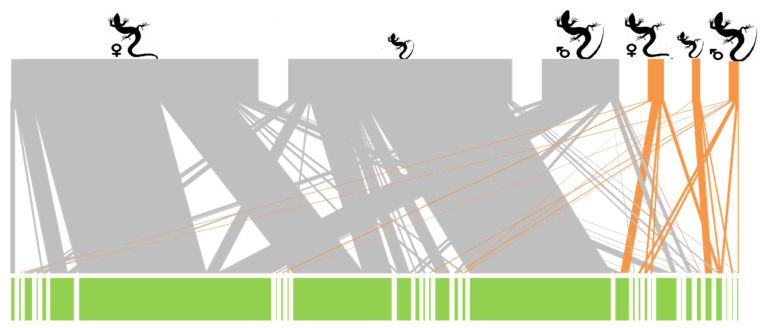
Interactions with flowers per lizard group observed from 5 April to 6 May 2022 on Cabrera Gran (Balearic Islands). Plant species are represented as green nodes, while lizards that visit the flower legitimately are grey nodes and those acting as florivores are orange nodes. Width of edges are proportional to the interaction rate.

**Figure 3:**
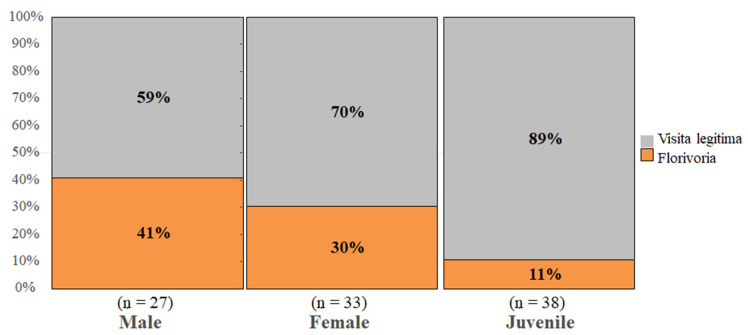
Relationship between the type of the interaction (legitimate visit vs. florivory) according to sex and age of lizards. Grey areas correspond to legitimate visits, while orange areas correspond to florivory.

Regarding sex, female lizards visited the flowers of a total of 26 plant species, while males interacted with 20 species and juveniles with 21 species (Figure 2). Florivory occurred in 9 (45%) species for males, 7 (27%) species for females and 3 (14%) species for juveniles. There was some overlap in flower use between the three groups although some flowering species were visited only by juveniles or by juveniles and females (Figure 2). *Globularia maritima* stands out in this sense: it was one of the flowers most visited by lizards but it only received visits from juveniles. We found a significant relationship between the type of interaction and the group of lizards (Chi^2^ = 8.18, d.f. = 2, *p* = 0.016; V_Cramer_ = 0.25, CI 95% [0.00, 1.00], n_obs_ = 98), with legitimate visits being more frequent than florivory for females (*p* = 0.02) and juveniles (*p* < 0.001). (Figure 3).

No direct observations of flower visits by geckos were recorded; however, one sample (n = 3) contained more than 100 pollen grains of a species that could not be identified.

## 4. Discussion

Balearic lizards have proven to be potential community-level pollinators by interacting with many different plant species to varying degrees. Although, in some plant species, lizards damaged reproductive structures by feeding directly on them, legitimate visits were significantly more frequent. Intraspecific differences were found in these wall lizards and even indications of gecko–flower interactions. These findings expand our knowledge not only in the magnitude of lizard–plant community interactions but also in their complexity.

Quantitatively, the lizards have a much higher frequency of interactions in plant species with flowers that appear very close together or are grouped in inflorescences with a certain density, such as *Daucus* sp. and *Lobularia* sp. (and therefore the contact with several flowers simultaneously is inevitable). In addition, on partially isolated rocks on steep shores, the flowers of plants such as *Daucus carota* attract the full attention of local lizards as they are often the only resource available in these habitats. This could, to some extent, overshadow the relevance of other plant species belonging to habitats of greater diversity and whose flowers are large and well isolated from each other, such as those of *Lavatera* spp. and *Cistus* spp.

Due to their relative abundance and generalist behavior, lizards seem to play an important ecological role in the reproduction of the plant community. A particular case is that of rockroses of the genus *Cistus*. In particular, *Cistus monspeliensis* is extremely abundant on the island of Cabrera and the legitimate interaction of lizards with its flowers, ignored until now, is one of the most important quantitatively. During the study, we also found the world’s largest population of Cartagena rockrose (*Cistus heterophyllus carthaginensis*), previously undescribed in the Balearic Islands [52]. It is a critically endangered species, whose threat factors include the biology of the species itself, which makes pollination difficult [53,54]. Its flower is very ephemeral (lasting only one day) and is not very attractive to insects. Although we observed lizards interacting with the flowers of this subspecies, the scarcity of available flowers prevented us from robustly quantifying the weight of this interaction.

The fact that some flowers are systematically bitten, and, in others, nectar is sipped by reptiles may be due to the characteristics of the flower. The sensitivity of lizards to be guided by odors is known [55], as is that different chemical compositions, for instance the presence to a lesser or greater extent of lipids, can generate specific responses, including the difference between sucking and biting [56,57,58]. Thus, chemical properties of flowers could be responsible for whether or not they are damaged by lizards. This may also justify why lizards ignore some species, since they have been reported to avoid plants that are poisonous to them as they are able to perceive the toxins [59]. On the smaller island of Na Redona, however, with less food available, scenes of intense florivory on *Medicago arborea*, *Lavatera maritima* and *Allium subvillosum* were observed, even climbing the branches or jumping directly from the ground and attacking flowers voraciously (pers. obs.). In this island, the Balearic lizard is a legitimate flower visitor of *Lavatera maritima* but can eat the flowers of this same species under dry conditions when other food resources are scarce [19]. Thus, it is also possible that the relationship between lizards and plants is as relevant as it is volatile in specific and delicate island habitats and that it is conditioned by a complex set of factors that deserve attention in future studies, such as food availability, the size of the island and environmental conditions, among others.

Despite this appreciation, analyses showed a significantly higher frequency of legitimate visits than florivory by female and juvenile lizards (Figure 3). It indicates that the use of flowers as a source of pollen/nectar is more important than the consumption of floral structures, and that lizards have the ability to directly access them. The Balearic lizard is not a herbivorous species, as this diet implies special adaptations [4] and reveals its outstanding relevance as a potential pollinator.

Males were the most florivorous group, showing no significant differences with legitimate visits. Male lizards have larger heads and therefore cannot access the floral cavities where the smallest ones (females and especially juveniles) can. The case of a female lizard that repeatedly visited a *Silene vulgaris* flower is illustrative, introducing its snout to feed inside it, while the male with which it shared territory was not interested in it. For larger lizards, sometimes the only alternative that allows them to obtain pollen and/or nectar from flowers is to break the structures by biting. However, it can also happen that, if these structures are not attractive and the reward is minimal, the flower does not arise sufficient interest. In this regard, we cite the case of *Cneorum tricoccon*, whose small yellow flowers are sucked by small lizards (juveniles and females) while the males focus their attention on the relatively large fruits. This mutualistic interaction is the most important [30] although the flowers are also consumed at least occasionally [39].

We know that reptiles [20,22], including lacertids [21], undergo ontogenetic changes in diet to adapt the size of the prey to that of the individual itself [28] and reduce intraspecific competition. This not only justifies the fact that the large males directly consume the complete flowers that the juveniles can access with their tongues by introducing their snouts into them, but also the exploratory tendency of the latter in small flowers to actively search for insects of a negligible size for the adults. This foraging behavior precisely is the reason why there is no overlap in some relevant plant species due to the intensity of interaction they receive, such as *Lobularia maritima*, only frequented by juveniles, and therefore different age classes or sexes can act as differentiated functional groups for the purposes of pollination. Juveniles can also show ethological differences associated not only with size but also with energy demand and ease of obtaining animal prey: as they are developing, they need a higher protein intake than adults and therefore have to invest more in foraging (search for small insects in small flowers) and less in the consumption of vegetable matter including floral structures (without the protein contribution provided by animal prey). The change in diet towards a higher vegetable fraction in adulthood is typical in generalist species of many families of lizards and is precisely positively associated with greater body size, not only because of the relative energy demand, but also because of the difficulty involved in feeding. Large prey are rarer and more difficult to obtain except for species from highly specialized predatory families, such as monitor lizards or chameleons, which can successfully capture them [26].

This fact was also reflected in the behavior of the Balearic lizards during hunting: on multiple occasions, the lizards made unsuccessful attempts to catch large insects such as diurnal lepidoptera, while small and low-mobility insects were caught effortlessly. A great need for food not covered by the availability of arthropods for large lizard males can lead them to feed more frequently on floral structures and also to visit fewer plant species, disregarding both those in which they do not have access to nectar for their body dimensions (and the structures themselves are not attractive), such as those whose only interest for lizards is the presence of small pollinating insects.

Like the Balearic lizards, geckos present intraspecific variations in diet [22], but unlike the former, they do not have such a broad trophic spectrum nor such a deep adaptation to Cabrera and this justifies their anecdotal relevance in the study. However, the seasonal variation in the diet of the common gecko reflects a certain opportunistic capacity [48,49,60], as well as the diversity of its behavior. Although it hunts on the vertical walls, precisely where it benefits from artificial lighting [61,62], it actively forages on the ground [49,63], thus being able to visit flowers. The lower risk of predation on islands has been suggested as an incentive for geckos to forage [64], as has the lizard’s tendency to expose itself by climbing on plants [65]. These points support that the gecko is revealed as a potential pollinator by using, at least sporadically, floral resources. Its survival on Cabrera, conditioned by the scarcity of insects, may also have led it to take advantage of the floral offer, either for direct consumption of nectar or for hunting other floral visitors. Although unprecedented, the finding is not surprising considering that visits to flowers by another congeneric species (*Tarentola delandii* in *Euphorbia lamarckii*) had already been discovered on islands in the Palearctic ecozone [66]. However, the importance of these geckos as potential pollinators should be much less relevant when compared to frequent floral visitors in the tropics such as *Phelsuma* sp. in Mauritius and Reunion [15,67,68,69]. In addition, the nocturnal activity of geckos makes their absence in the censuses logical. A night sampling work with special cameras for this purpose could reveal more information on this matter.

## 5. Conclusions

The discovery of new interactions with flowers for the Balearic lizard—a species that has received much attention so far [19]—demonstrates that this mutualism is greatly underestimated concerning its real magnitude. In this sense, extending the observational effort to other species not considered, such as those that flower at other times of the year, may continue to provide new findings. This result also justifies the need for further studies at the community level, quantifying the weight of interactions and comparing the role of lizards with other potential pollinators. In the case of geckos, more night sampling is needed to collect more information on flower use by these species.

## Figures and Tables

**Table 1:** Interactions between the Balearic lizard (*Podarcis lilfordi*) and flowers observed from 5 April to 6 May 2022 on Cabrera Gran (Balearic Islands). Note: four morphospecies found in pollen samples could not be identified.

Plant Species	Interaction Type	Known on Cabrera Gran	Known Anywhere
*Apiaceae*	Unknown (pollen samples)	No	No
*Asparagus horridus*	Legitimate visit	No	No
*Asphodelus fistulosus*	Florivory	No	No
*Astragalus balearicus*	Florivory	No	No
*Calendula arvensis*	Legitimate visit	No	Aire, Rei, Sanitja [47]
*Cakile maritima*	Florivory	No	No
*Centrathus calcitrapae*	Legitimate visit	No	Na Redona [Islet food-web team; pers. observ.]
*Cistus heterophyllus*	Legitimate visit	No	No
*Cistus monspeliensis*	Legitimate visit	No	No
*Daucus carota*	Legitimate visit	No	No
*Echium parviflorum*	Legitimate visit	No	No
*Erodium malacoides*	Florivory	No	No
*Ephedra fragilis*	Legitimate visit	Yes [34]	Na Redona, Dragonera [Islet food-web team; pers. observ.]; [34]
*Euphorbia dendroides*	Legitimate visit	Yes [5]	Na Redona [Islet food-web team; pers. observ.]
*Euphorbia segetalis*	Legitimate visit	No	No
*Fumana ericoides*	Legitimate visit	Yes [41]	No
*Geranium molle*	Legitimate visit	No	Na Redona [Islet food-web team; pers. observ.]
*Helichrysum stoechas*	Florivory	No	Estell de dos Colls [41]
*Lavatera arborea*	Legitimate visit	No	Xes Rates, Xapat Gros [41]
*Lavatera cretica*	Florivory	No	No
*Linum* sp.	Florivory	No	No
*Lobularia maritima*	Legitimate visit	No	No
*Lomelosia cretica*	Legitimate visit	No	No
*Lysimachia arvensis*	Legitimate visit	No	No
*Medicago arborea*	Legitimate visit	No	Estell de dos Colls, Na Redona [41]; [Islet food-web team; pers. observ.]
*Medicago littoralis*	Florivory	No	Aire, Rei, Sanitja [47]
*Melilotus* sp.	Florivory	No	No
*Narcissus tazetta*	Legitimate visit	No	Rei [47]
*Papaver somniferum*	Florivory	No	No
*Paronychia capitata*	Legitimate visit	No	No
*Plumbago* sp.	Legitimate visit	No	No
*Rubia peregrina*	Legitimate visit	No	No
*Ruta graveolens*	Legitimate visit	No	No
*Rhamnus ludovici-salvatoris*	Legitimate visit	No	No
*Salvia rosmarinus*	Legitimate visit	No	Menorca [45]
*Scorpiurus sulcatus*	Florivory	No	No
*Silene vulgaris*	Legitimate visit	No	No
*Sinapis arvensis*	Florivory	No	No
*Sonchus tenerrimus*	Florivory	No	No
*Teucrium capitatum*	Legitimate visit	No	No

## Data Availability

The data presented in this study are available on request from the corresponding author.

## Supplementary Materials

The following supporting information can be downloaded at: https://www.mdpi.com/article/10.3390/ani13061122/s1, List of all plant species censused to detect lizard–flower interactions, from 5 April to 6 May 2022 on Cabrera Gran (Balearic Islands).
